# Comparing the Dimensional Accuracy of Casts Obtained from Two Types of Silicone Impression Materials in Different Impression Techniques and Frequent Times of Cast Preparation

**DOI:** 10.1155/2021/9977478

**Published:** 2021-09-27

**Authors:** Ali Hafezeqoran, Mahdi Rahbar, Roodabeh Koodaryan, Tina Molaei

**Affiliations:** ^1^Department of Prosthodontics, School of Dentistry, Tabriz University of Medical Sciences, Tabriz, Iran; ^2^Dental and Periodontal Research Center, Faculty of Dentistry, Tabriz University of Medical Sciences, Tabriz, Iran; ^3^Department of Esthetic and Restorative Dentistry, School of Dentistry, Ardabil University of Medical Sciences, Ardabil, Iran; ^4^Students Research Committee, School of Dentistry, Ardabil University of Medical Sciences, Ardabil, Iran

## Abstract

**Introduction:**

The dimensional accuracy of casts is essential in the quality of fixed prosthesis treatment, whereby the impression method is a very crucial factor affecting it. The aim of this in vitro study is to compare the dimensional accuracy of casts resulting from two types of silicone impression materials in different impression techniques and frequent times of cast preparation.

**Materials and Methods:**

A metal model was made from two prepared abutments, and 10 casts were prepared from each material technique (*n* = 40). The impressions were made by condensation and addition silicone (one-stage and two-stage impressions). The casts were made from same impressions 1 h, 24 h, and 14 days. The diameter, height, and the distance between two dies were recorded. Data were analyzed by repeated measure ANOVA (*P* value <0.05).

**Results:**

The dimensional accuracy of all four materials techniques of impression (diameter, height, and the distance between dies) was the same in different times of impression. Dimensional accuracy of the die diameter and distance between dies in one-stage (Speedex) condensation silicon and one-stage (Panasil) addition silicone did not differ significantly, and their one-stage method developed more accurate casts compared to the two-stage method of the same impression material. The height of the casts prepared from the one-stage method through Speedex and Panasil did not differ significantly from the two-stage method of the same impression material.

**Conclusion:**

One-stage condensation silicone and one-stage addition silicone material techniques offered the maximum dimensional accuracy in the obtained casts. The time of impression did not have any significant effect in the accuracy of any of the four impression material techniques.

## 1. Introduction

An accurate impression will result in accurate dental marginal adaptation of casting restorations and eventually contribute to the longevity of the restoration. On the other hand, marginal gap in this stage results in a prosthesis with improper adaptation. One important group of impression materials is elastomers, among which polyethers and silicons (condensation and addition) are more common in fixed prosthesis impression [1–6].

The ability of preparing various casts from a developed impression is clinically important [7, 8]. In many cases, the need to renew the cast is due to problems on the first cast or due to the laboratory steps. Under these conditions, a good material can pour the impression prepared and made several casts; and the accuracy of the primary cast is required [1].

Thongthammachat et al. investigated the effect of impression pouring time of addition silicone and polyether impression materials within the time intervals of 30 min, 6 h, and 24 h following impression. They found that the polyether impressions are better to be pouring only once and up to 24 h following impression due to deformation over time. However, the addition silicone impression has a better dimensional stability compared to polyether and can even maintain it up to 30 days [9]. In the investigation by Johnson and Craig, it was also found that in terms of repouring, addition silicone and condensation silicone do not show any difference in casts, but changes in polysulfide and polyether are noticeable [10].

In a study conducted by Pandita et al. on addition silicone and vinyl polyether silicon, they concluded that in repouring at 1 h, 24 h, and 14 days following impression, the dies made of vinyl polyether were smaller at all three times. The dies made from addition silicone were smaller after 1 h and 24 h, yet larger after 14 days. Clinically, the extent of changes in both impression materials lied within an acceptable range [11].

Torabi et al. found that Correct Plus and Panasil addition silicones can be used to make four different casts with almost the same accuracy at time intervals of 30 min, 90 min, 150 min, and 24 h. However, Speedex condensation silicone impression could be used to make at most three casts at time intervals of 30, 90, and 150 min. On the other hand, according to Tuit and Rosen, two accurate casts with 1 h time interval can be made from addition impression materials, different from the findings of the mentioned study [12].

The aim of this study is to investigate the dimensional accuracy of casts resulting from two types of silicone impression materials (condensation silicone/addition silicone) at three different times (and using two different techniques, one-stage and two-stage impressions).

## 2. Materials and Methods

In this in vitro study, condensation silicone (Alstatten, Coltene, Speedex, Switzerland) and addition silicone (Panasil, Kettenbach, Eschenburg, Germany) were investigated.

**Figure 1:**
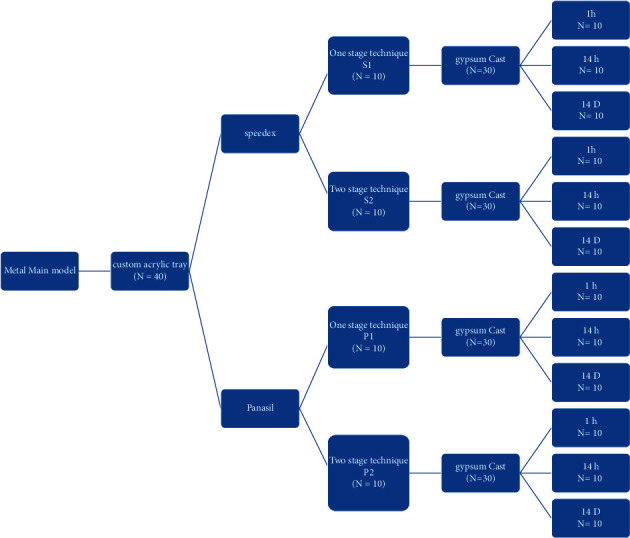
The study design (the materials, times, and techniques).

To determine the sample size, power and sample size software (developers: W.D. Dupont and W.D. Plummer, country: United States) was used along with similar studies [11, 13]. Considering *α* = 0.05, power of 80%, and mean and standard deviation of 0.3 ± 0.2, respectively, eight samples were estimated to measure the variable of dimensional accuracy in each material technique. However, to enhance the accuracy of this study, 10 impression samples were considered for each group (*n* = 10), and an overall 40 impression samples was considered for four different material techniques, as shown in Figure 1.

**Figure 2:**
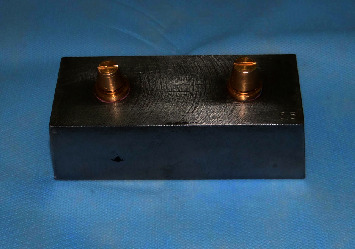
The main model used in this study to investigate the dimensional accuracy of the impression materials.

The model employed for measuring the dimensional accuracy (Figure 2) had two metal dies as a cone frustum, as with two prepared teeth, which had cervical diameters of 11 and 13.72 mm.

**Figure 3:**
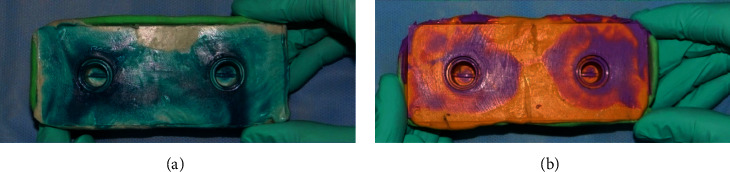
(a) The impression prepared from Speedex as two-stage (*S*2); (b) the impression prepared from Panasil as one-stage injection (*P*1).

Each of them had a convergence angle of 6°, situated 47 mm away from a fixed metal base with 14 mm high (as with the bases of a long bridge). To develop space in the tray, 6 mm thick wax was used. Eventually, 40 custom acrylic trays (10 for each technique) (Triplex Hot, Ivoclar Vivadent Inc., Lichtenstein, Germany) were made with suitable path of insertion and support. The trays were made in such a way to prevent them from moving in vertical and horizontal dimensions during impression. Then, according to the manufacturer's instructions, impression was performed from the model using custom trays and impression materials (Figure 3).

**Figure 4:**
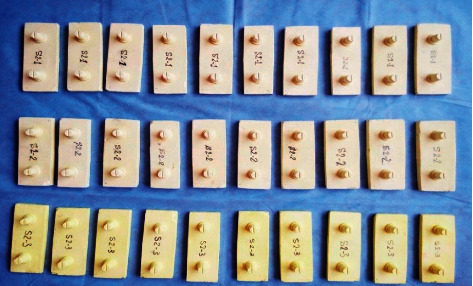
The casts prepared from two-stage Speedex impression at different times including 1 h, 24 h, and 14 days after impression (10 samples for each studied time).

After complete polymerization and recovery time of the elastic phase, the casts were made with type IV dental gypsum (Ernst Hinrichs GmbH, Goslar, Germany). The gypsum was mixed based on the powder and water ratio recommended by the manufacturers factory, and the final mixing was performed automatically (Multirac4, Degussa AG, Frankfurt, Germany) for at least 20 s under vacuum, and pouring was performed with slow vibration. The castes were separated from the tray 45 minutes after pouring. All impressions were made and kept at room temperature (23°C). Also, plaster samples were also prepared and kept at room temperature (23°C) to make the uniform environmental condition. The first, second, and third plaster samples were casted after 1 h, 24 h, and 14 days after impression [11]. For each impression material and technique, 10 casts were made (totally 40 casts including 10 casts with each of the Speedex and Panasil impression materials using the one-stage putty-wash method (*S*1, *P*1) and 10 casts from each of them through the two-stage putty-wash method (*S*2, *P*2) (Figure 4 and Table 1)).

Three casts were made from each impression, and overall, 120 plaster casts were prepared from the four different material techniques. The samples were examined by a magnifier with 3X magnification, and the samples which had cracks, fracture, bubbles, and any other structural problems were excluded from the study.

**Figure 5:**
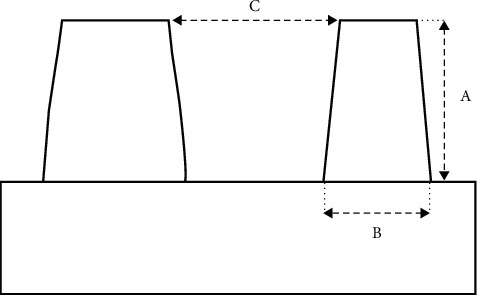
The schematic view of the measured dimensions in the plaster models related to investigating the dimensional accuracy of the casts obtained from different impression methods. (a) The height of the small die in the external part. (b) The width (diameter) of the small die in the base. (c) The distance between the internal vertices of dies with each other.

In this study, to measure the dimensions of models, the stereomicroscope (P-IBSS2, Nikon, Japan) was used with the accuracy of 0.001 mm, with a digital caliper (Guanglu Digial Caliper, China) with the accuracy of 0.01 mm according to ANSI/ADA No. 25 standard [4]. The stereomicroscope was used to measure the height and diameter of the die, and the digital caliper was employed to measure the distance between two dies. In order to compare the plaster samples with the main metal die, the measurement was performed in three dimensions (height, width, and distance between internal vertices) (Figure 5).

In order to control parameters, the measurements were performed by a blinded examiner. Each of the measurements was repeated three times. Data obtained were tested by repeated measures ANOVA, and the post hoc Tukey test (significant level) was used.

## 3. Results

The mean ± standard deviation of the diameter of the small die, height of die, and distance of the internal vertex of dies based on different impression methods and at different times of pouring are given in Table 2.

**Figure 6:**
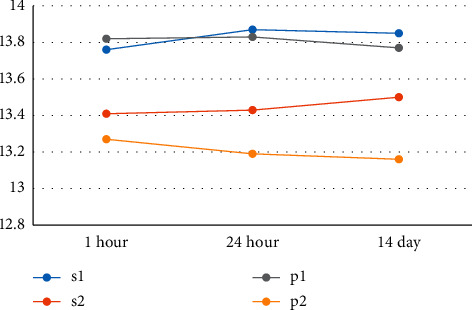
The mean diameter of die based on different impression methods and different times of cast preparation (1 h, 24 h, and 14 days after impression).

**Figure 7:**
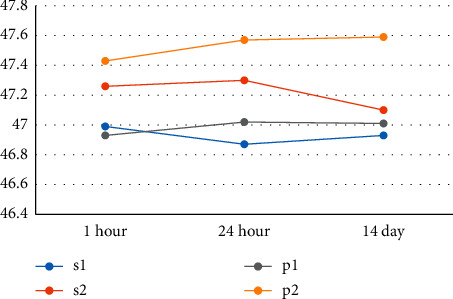
The mean distance between the inner vertex of the dies with each other based on different impression methods and different times of cast preparation (1 h, 24 h, and 14 days after impression).

Investigation of the mean diameter of die and distance of internal vertex of dies with each other based on different impression methods and at different times of cast preparation (Figures 6 and 7) indicates that the die diameter and the distance of internal vertex diameter of dies with each other are closer to the main model in the casts resulting from impression using one-stage Speedex (*S*1) and one-stage Panasil (*P*1) techniques, when compared with two-stage Speedex (*S*2) and two-stage Panasil (*P*2) techniques.

According to Table 3 (the results of the one-way ANOVA analysis), comparison of mean diameter of the die and the internal vertex distance of dies with each other based on different impression methods and at different times of cast preparation indicates that there is no significant difference between groups.

There is no significant difference between the mean diameter of die in one-stage Speedex casts one hour later, one day later, and two weeks later (*P* value_die diameter_ = 0.96 and *P* value_internal vertex distance of dies_ = 0.85). On the other hand, this test shows a significant difference between groups (*P* value = 0.001). It was found that there is a statistically significant difference between the mean diameter of die and internal vertex distance of dies with each other in the paired groups of *S*1 with *S*2, *S*1 with *P*2, *P*1 with *P*2, *P*1 with *S*2, and eventually *S*2 with *P*2, and with all paired groups under investigation (*P* value <0.05). Indeed, the dimensional accuracy of the mean diameter of die and internal vertex distance of dies with each other are almost the same at different times of impression pouring (cast preparation) across all impression techniques utilized in this study. Furthermore, there was no significant difference between the mean diameter of die in the material techniques of one-stage Speedex and one-stage Panasil. The dimensional accuracy of the mean diameter of die and the internal vertex distance of dies is higher in the casts resulting from one-stage impression through Speedex and Panasil compared to the two-stage method of the same impression material.

**Figure 8:**
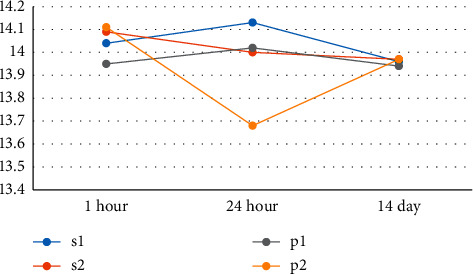
The mean height of die based on different impression methods and different times of cast preparation (1 h, 24 h, and 14 days after impression).

Investigation of the mean height of die based on different impression methods and different times of impression pouring according to Figure 8 indicates that the mean height of die has been closer to the die height of the main model in the casts resulting from one-stage Speedex (*S*1), two-stage Speedex (*S*2), and one-stage Panasil (*P*1) techniques.

The results of the one-way ANOVA test to compare height of die also showed a significant difference between and within groups (_between-subjects_*P* value = 0.09; _within-subjects_*P* value = 0.06) The results show that there is no significant difference between the dimensional accuracies of die height in different material techniques. The dimensional accuracy of the mean die height resulting from one-stage impression by Speedex and Panasil has not been significantly different compared to the two-stage method of the same impression material.

## 4. Discussion

Undoubtedly, one of the most important stages of treatment in fixed prosthesis is accurate impression, which determines the success or failure and prognosis of the treatment. Neglecting this stage treatment will lead to an inaccurate plaster cast and eventually a prosthesis with improper adaptation. In case of inaccuracy, the impression should be repeated, spending costs and time. Therefore, selecting the best and most accurate impression method is essential for successful treatment [14].

The results of the present research indicated that among the four studied material techniques of impression, there was no significant difference between the dimensional accuracy of the mean diameter and height of small die and the internal vertex distance of dies at different times of impression pouring. Furthermore, the dimensional accuracy of the mean diameter of the die and the internal vertex distance of dies with each other in the casts obtained from one-stage Speedex and one-stage Panasil material techniques have been almost the same. On the other hand, the dimensional accuracy in the casts obtained from one-stage impression with Speedex and Panasil has been greater than that of the two-stage of the same impression material.

The mean height of die in the casts prepared in different material techniques did not have any significant difference at different times. Also, there was no significant difference between one-stage impression (by Speedex and Panasil) and the two-stage of the same impression material.

Levartovsky et al. examined the effect of one-stage and two-stage putty-wash impression methods on long-term dimensional accuracy and stability of polyvinyl siloxane (PVS). They found that when using the two-stage putty-wash impression method, impressions pouring can be delayed up to 30 h [15]. Similarly, in our research, two-week delay did not significantly affect the dimensional accuracy. In the mentioned study, the two-stage impression technique showed greater accuracy compared to the one-stage method. On the other hand, according to the present research, the one-stage method showed greater accuracy. The reason of the difference in the results is possibly due to the fact that in the study by Levartovsky et al., dimensional accuracy measurement had been performed only in one molar tooth and eventually six days following impression. However, in the present study, impression from fixed bridge was performed, and up to 14 days after impression, the accuracy was measured.

In another study conducted with the aim of investigating the effect of the impression method and repouring on the accuracy of casts using addition silicone, the ANOVA test showed that there is no significant relationship between impression methods and the accuracy of the first cast resulting from the impressions with the main model [8]. Similarly, in the present study, nonsignificant changes in the dimensions of the first casts obtained from different techniques were demonstrated.

The findings obtained by Avila et al. in comparing the dimensional accuracy resulting from PVS impression indicated that there is no significant difference between the casts obtained from this impression material [16]. On the other hand, according to the present study, the dimensional accuracy of the mean diameter of the die and the internal vertex distance of dies with each other in the one-stage impression by PVS has been higher compared to the two-stage method of the same impression material. The reason is due to the different methodologies of two studies. In the mentioned study, first, a prefabricated tray had been used, and the number of samples considered for each method was half of the sample numbers in the present study (*n* = 5). Furthermore, to investigate the dimensional accuracy, only the gap between the framework and abutment had been considered.

Dugal et al. in an in vitro study compared the dimensional accuracy of casts resulting from one-stage and two-stage PVS techniques. Based on this study, the two-stage method had the maximum dimensional accuracy among the casts [17], which is in contrast to our results. The different commercial brand and study sample numbers can be the reasons of this difference.

Nili Ahmadabadi et al. investigated the effect of second wash on the dimensional accuracy of plaster casts resulting from one-stage and two-stage impression methods with spacing with Speedex impression material. Unlike the results of the present study, they observed that the two-stage method was more accurate than the other methods. They also reported that the one-stage method with second wash was more accurate than the one-stage method alone. Furthermore, they found usage of the two-stage method with second wash inappropriate for the bridges [14]. In the study by Nili Ahmadabadi, no comparison had been made between different impression materials, and they only dealt with examining primary casts. In the present study, we decided to use both condensation silicone and addition silicone and investigate the effect of time in preparation of plaster casts from the initial impression.

Another study with the aim of comparing two impression techniques (two-stage with and without spacer) was performed using addition silicone on an experimental model. In both techniques, the die height had been diminished, but the difference was not significant [18]. These results are consistent with the current study. The die diameter had changed in the two-stage method and was significant. In terms of distance between two dies, the two-stage impression technique with a spacer had very minor variations [18]. However, in the present research, the one-stage method showed the minimum changes in relation to the main model.

In another study performed on Speedex impression material, it was reported that in one-stage and two-stage Speedex impression techniques without a spacer, there is no difference in terms of die unlike the present study. Also, the height of die in the two-stage method without a spacer was more accurate than the one-stage technique [19], which is in line with the results of the present study. Comparison of the three impression methods with polyvinyl siloxane on the extent of die dimensional variations indicated that the impression methods did not have any effect on the die dimensional changes or the extent of these changes is negligible [20]. Nevertheless, in this study, the effect of time had not been inspected on the accuracy of the plaster casts. On the other hand, based on the present research, the one-stage method showed greater accuracy compared to the two-state method at three different times ranges.

In a study comparing the dimensional accuracy and stability of Speedex and Irasil impression materials belonging to the group of condensation silicons, it was found that the time was not influential in any of the studied materials with regards to dimensions of the casts obtained [21]. In the present research, a similar result was obtained, and it was found that the dimensional accuracy of die height and internal vertex distance of dies with each other are not affected by time in any of the impression techniques using Speedex.

Investigation of the dimensional accuracy of two commercial brands of condensation silicone using the putty-wash method showed that the die height decreased in both studied types. However, according to the statistical test, there was no significant difference between the two groups and main model, which is in line with our study [22].

In another research conducted to determine the dimensional accuracy of three putty-wash impression methods by polyvinyl siloxane, among the putty-wash impression methods with polyvinyl siloxane, the two-stage method with a spacer was introduced as the most accurate method for making casting restorations [23]. However, in our study, the one-stage method was considered as the best impression method.

This investigation was performed experimentally. In our experimental method, despite development of suitable conditions, mouth conditions (in terms of saliva, blood, and temperature) are not established. In addition, in this investigation across the measured die dimensions, the effect of confounding factors such as making wax pattern and type of casting metal has not been examined. Thus, it is suggested that the effect of mentioned impression materials be examined on the extent of restoration leakage.

## 5. Conclusions

Generally, based on the obtained information from this study, the following conclusions can be drawn:

1. The dimensional accuracy of the mean diameter and height of small die and the internal vertex distance of dies with each other at different times of impression pouring have been almost the same in all utilized impression techniques. There is no significant difference between various times of impression pouring.
2. The difference of the dimensional accuracy of the mean diameter of the die and internal vertex distance of dies with each other was not significant between the one-stage Speedex and one-stage Panasil material techniques
3. The dimensional accuracy of the mean diameter of the die and internal vertex distance of dies with each other was not significant between the casts obtained from the one-stage impression method by Speedex and Panasil compared to the two-stage method of the same impression material.
4. There is no significant difference between the dimensional accuracy of die height in different material techniques.

## Figures and Tables

**Table 1:** Materials used in this study.

Material	Composition	Manufacturer
Impression materials	Condensation silicone impression	Alstatten, Coltene, Speedex, Switzerland
	Addition silicone impression	Panasil, Kettenbach, Eschenburg, Germany

Dental gypsum	Type IV gypsum	Ernst Hinrichs GmbH, Goslar, Germany

**Table 2:** The mean dimensional accuracy measured based on different impression methods (one-stage or two-stage) and different times of impression preparation (1 h, 24 h, and 14 days after impression).

Dimensional accuracy measurement time	Impression method	Small die diameter (SD ± mean)	Die height (SD ± mean)	Distance between the inner vertices of the dies (SD ± mean)
Dimensional accuracy measured after one hour	*S*1	0.14 ± 13.76	0.12 ± 14.04	0.32 ± 46.99
	*S*2	0.12 ± 13.41	0.33 14.09±	0.29 47.26±
	*P*1	0.09 ± 13.82	0.07 ± 13.95	0.15 ± 46.93
	*P*2	0.17 ± 13.27	0.18 ± 14.11	0.19 ± 47.43

Dimensional accuracy measured after a day	*S*1	0.12 ± 13.87	0.21 ± 14.13	0.09 ± 46.87
	*S*2	0.15 ± 13.43	0.13 ± 14.00	0.35 ± 47.300
	*P*1	0.10 ± 13.83	0.06 ± 14.02	0.07 ± 47.02
	*P*2	0.21 ± 13.19	0.53 ± 13.68	0.20 ± 47.57

Dimensional accuracy measured after two weeks	*S*1	0.17 ± 13.85	0.19 ± 13.96	0.43 ± 46.93
	*S*2	0.23 ± 13.50	0.23 ± 13.97	0.27 ± 47.10
	*P*1	0.07 ± 13.77	0.12 ± 13.94	0.10 ± 47.01
	*P*2	0.20 ± 13.16	0.17 ± 13.97	0.21 ± 47.59

Small die diameter size on the model: 13.72 mm; die height size on the model: 14.00 mm; distance between the inner vertices of the dies size on the model: 47.00 mm. SD, standard deviation; *S*1, Speedex one-stage technique; *P*1, Panasil one-stage technique; *S*2, Speedex two-stage technique; P2, Panasil two-stage technique.

**Table 3:** The results of one-way ANOVA about the mean diameter of the small die, die height, and the internal vertex distance of dies based on different impression methods (one-stage or two-stage) and different times of cast preparation (1 h, 24 h, and 14 days after impression).

Source of changes	*P* value die diameter	*P* value die height	*P* value internal vertex distance of dies
Between-subjects	0.00	0.09	0.00
Within-subjects	0.96	0.06	0.85

## Data Availability

The data used to support the findings of this study are included within the article.
